# Nanostructure selenium compounds as pseudocapacitive electrodes for high-performance asymmetric supercapacitor

**DOI:** 10.1098/rsos.171186

**Published:** 2018-01-10

**Authors:** Guofu Ma, Fengting Hua, Kanjun Sun, Enke Fenga, Hui Peng, Zhiguo Zhang, Ziqiang Lei

**Affiliations:** 1 Key Laboratory of Eco-Environment-Related Polymer Materials of Ministry of Education, Key Laboratory of Polymer Materials of Gansu Province, College of Chemistry and Chemical Engineering, Northwest Normal University, Lanzhou 730070, People's Republic of China; 2 College of Chemistry and Environmental Science, Lanzhou City University, Lanzhou 730070, People's Republic of China

**Keywords:** Bi_18_SeO_29_/BiSe, Co_0.85_Se, asymmetric supercapacitor

## Abstract

The electrochemical performance of an energy conversion and storage device like the supercapacitor mainly depends on the microstructure and morphology of the electrodes. In this paper, to improve the capacitance performance of the supercapacitor, the all-pseudocapacitive electrodes of lamella-like Bi_18_SeO_29_/BiSe as the negative electrode and flower-like Co_0.85_Se nanosheets as the positive electrode are synthesized by using a facile low-temperature one-step hydrothermal method. The microstructures and morphology of the electrode materials are carefully characterized, and the capacitance performances are also tested. The Bi_18_SeO_29_/BiSe and Co_0.85_Se have high specific capacitance (471.3 F g^–1^ and 255 F g^–1^ at 0.5 A g^–1^), high conductivity, outstanding cycling stability, as well as good rate capability. The assembled asymmetric supercapacitor completely based on the pseudocapacitive electrodes exhibits outstanding cycling stability (about 93% capacitance retention after 5000 cycles). Moreover, the devices exhibit high energy density of 24.2 Wh kg^–1^ at a power density of 871.2 W kg^–1^ in the voltage window of 0–1.6 V with 2 M KOH solution.

## 1. Introduction

Supercapacitors, prospective energy storage and conversion devices, have attracted tremendous interest due to the need for power output devices for digital communications and electric vehicles owing to their facile manufacture, fast charging/discharge, long cycle life and higher power density than batteries [1–4]. Intrinsically, supercapacitors based on the principle of charge storage are divided into two types: pseudocapacitors and electric double-layer capacitors (EDLCs) [5]. In EDLCs, the charges are stored because of the surface adsorption of the ions from the electrolyte as a result of electrostatic attraction, thus forming two charged layers (double layer). Pseudocapacitors store charges by fast and reversible oxidation/reduction (Faradaic) reactions occurring at the electrode/electrolyte interfaces, as well as in the bulk near the surface of the electrode. Pseudocapacitors show higher capacitance when compared with EDLC-type devices due to the additional charge transferred within the defined potential [6–8].

Up to now, electrode materials with long cycle stability and high capacitance have obtained breakthrough progress, for instance transition metal sulfides (CuS [9], FeS [10], Al_2_S_3_ [11], etc.), transition metal oxides/hydroxides (NiO [12], ZnO [13], TiO_2_ [14], Co(OH)_2_ [15], CoNi_2_S_4_ [16], etc.) as well as conducting polymer materials (polyaniline [17], polypyrrole [18], etc.). The above electrode materials have been investigated for use in asymmetric supercapacitors extensively, due to their fast reversible redox reactions, cost-effectiveness, easy processability and relatively good cyclic stability [19]. However, compared with the transition metal dichalcogenide, the two-dimensional (2D) layered transition metal selenide has been paid less attention. The weak van der Waals force of 2D layered metal selenide is beneficial for insertion of guest species. Owing to their superior electronic structure and physical properties, the 2D nanosheet structural materials have attracted abundant attention compared to the corresponding bulk materials [20]. Recently, transition metal selenide has been shown to display outstanding electrochemical performance. Balasingam *et al*. have synthesized a few-layered MoSe_2_ nanosheet by a simple hydrothermal method and further studied its electrochemical charge storage properties. It is shown that the MoSe_2_ nanosheet electrode provides a symmetric device that exhibits 49.7 F g^–1^ with a scan rate of 2 mV s^–1^ and a largest specific capacitance of 198.9 F g^–1^ [21]. Wang *et al*. made a flexible all-solid-state supercapacitor based on three-dimensional (3D) hierarchical GeSe_2_ nanomaterials, which have a high specific capacitance of 300 F g^−1^ at a current density of 1 A g^–1^[22]. Choi *et al*. synthesized SnSe_2_ nanoplate–graphene composites and used them as a novel anode material for lithium ion batteries [23]. Tang *et al*. synthesized a new type of binder-free electrode material of NiSe/NF (NiSe nanowire film on nickel foam) by using the one-step hydrothermal method; it has a high specific capacitance of 1790 F g^–1^ at a current density of 5 A g^–1^ [24]. Yang *et al*. developed interconnected Co_0.85_Se nanomaterials on nickel foam directly through a facile single-step hydrothermal method, which exhibits a high specific capacitance of 1528 F g^–1^ at 1 A g^–1^ and excellent cycling stability [25]. Therefore, the transition metal selenide possesses outstanding electrochemical energy storage properties and deserves to be further investigated as an advanced electrode material for supercapacitor application.

In recent years, in order to improve energy density and power density to attain a win–win situation, researchers have assembled asymmetric supercapacitors (ASCs) with different electrode materials in aqueous electrolytes [26–30]. According to the literature, *E* = 1/2*CV*^2^ (energy density formula); in order to improve energy density (*E*), two approaches can be used: maximizing the specific capacitance (*C*) and/or enlarging the operating potential window (*V*). Employing ionic liquids or organic electrolytes can increase the operating voltage effectively. However, their inherent deficiencies such as poor ionic conductivity and sometimes toxicity have hindered their practical application [26,27]. In comparison, an aqueous electrolyte is the best choice. The ASC is made up of two different electrodes, hence it is an essential prerequisite to select appropriate positive and negative electrode materials to assemble a high-performance ASC. Recently, the literature has depicted that the transition metal selenide has excellent electrochemical performance for a supercapacitor. But the synthesis of different selenium compounds as the positive and negative electrodes of a supercapacitor in the same system has not been well explored so far. In addition, the benzyl alcohol route in particular has been proved to be versatile for the synthesis of various metal oxide nanoparticles with good control over particle phase, size and shape [28,29]. The fact that the benzyl alcohol route is typically applied without the use of surfactants makes this approach ideal for mechanistic studies. Additives such as surfactants complicate the interpretation of the results due to their possible influence on nucleation and growth by complexation of cations [30,31]. Therefore, in a two-component system just consisting of a precursor and solvent, the complexity is decreased to a minimum, although benzyl alcohol itself may play multiple roles as reaction medium, oxygen source and/or capping agent. Another unique feature of non-aqueous systems is the possibility to monitor the organic reactions occurring in parallel to nanoparticle formation by standard analytical techniques. Thus, the use of benzyl alcohol as a solvent (without any template and surfactant) to synthesize transition metal selenium-based compounds is a very desirable route.

In this work, we have used a facile low-temperature one-step hydrothermal method without any template and surfactant (benzyl alcohol as the solvent) to synthesize two different transition metal selenides: Bi_18_SeO_29_/BiSe and Co_0.85_Se. The lamella-like Bi_18_SeO_29_/BiSe and petal-like Co_0.85_Se, which are used as negative and positive electrode materials, have high specific capacitance in aqueous electrolyte. The assembled ASCs possess excellent energy density and outstanding power density with a wide voltage window as well as high cycling stability in aqueous electrolyte.

## 2. Experimental

### 2.1. Materials

Bismuth nitrate pentahydrate (Bi(NO_3_)_3_•5H_2_O, Aladdin Industrial corporation, Shanghai, China), cobalt nitrate hexahydrate (Co(NO_3_)_2_•6H_2_O, Aladdin Industrial corporation, Shanghai, China), selenium oxide (SeO_2_, Sitong Tianjin Chemical Reagent Co. Ltd, China) and benzyl alcohol (Aladdin Industrial corporation, Shanghai, China). All chemical reagents were of analytical grade and were not further purified before use.

### 2.2. Synthesis of Bi_18_SeO_29_/BiSe nanocomposite

The Bi_18_SeO_29_/BiSe nanocomposites were synthesized using the hydrothermal method in benzyl alcohol. In the typical process, Bi(NO_3_)_3_•5H_2_O (0.582 g) and SeO_2_ (0.111 g) were dispersed in benzyl alcohol solution (35 ml), stirring uniformly for 1 h with the assistance of ultrasonic vibration. After stirring vigorously at room temperature for about 10 min, the white solution was transferred to a 50 ml Teflon-lined stainless steel autoclave and heated at 180°C for 15 h. Finally, the resulting greyish precipitate was collected by centrifugation and on cooling to room temperature naturally. Subsequently, the greyish precipitate was washed with distilled water and ethanol several times to remove any possible ions and dried in a vacuum at 65°C overnight.

### 2.3. Synthesis of Co_0.85_Se nanomaterials

The cobaltous selenide (Co_0.85_Se) nanosheets may also be synthesized by the hydrothermal method in benzyl alcohol solution; using 0.349 g Co(NO_3_)_2_•6H_2_O and 0.111 g SeO_2_ as raw materials, the process was similar to the synthesis of Bi_18_SeO_29_/BiSe nanocomposites.

### 2.4. Materials characterization

The morphologies of Bi_18_SeO_29_/BiSe and Co_0.85_Se were analysed using field emission scanning electron microscopy (SEM, Ultra Plus, Carl Zeiss) with a voltage of 5.0 kV. The microstructure of Bi_18_SeO_29_/BiSe and Co_0.85_Se were further characterized by transmission electron microscopy (TEM, JEM-2010 Japan). The crystallographic structure of the materials was degassed at 200°C before nitrogen adsorption measurement. X-ray diffraction (XRD) was conducted using a Rigaku D/Max-2400 diffractometer, with Cu Ka radiation (*λ* = 0.15418 nm) at 40 kV and 100 mA. X-ray photoelectron spectroscopy (XPS) was performed using an Escalab 210 system (Germany). In the SEM, the elemental mapping of relative energy-dispersive X-ray spectrometry (EDS) was performed by a probe focused to 0.2 nm with a camera length of 20 cm.

### 2.5. Electrochemical measurements

#### 2.5.1. Three-electrode system

The electrochemical performance of Bi_18_SeO_29_/BiSe and Co_0.85_Se was studied in 2 M KOH solution using a three-electrode system. High-purity carbon rods and oxidation of saturated mercury electrode (Hg/HgO) were used as the counter electrode and the reference electrode, respectively. The working electrodes were manufactured as follows: in general, 80 wt% electrode materials (16.0 mg), 10 wt% commercial carbon black (acetylene black, 2.0 mg) as well as 10 wt% polymer binder (polyvinylidene fluoride, 2 mg) were mixed with some *N*-methyl-2-pyrrolidone to form a homogeneous slurry. Further, the obtained slurry was coated on nickel foam with an area of 1.0 cm^2^ and dried at 65°C overnight, and then weighed and pressed into sheets under 13 MPa to ensure adherence between the active materials and the current collector. The total mass of each electrode was limited to 3.0–5.0 mg [32].

#### 2.5.2. Two-electrode system of asymmetric supercapacitors

The electrochemical measurements were further taken using a two-electrode system consisting of Bi_18_SeO_29_/BiSe and Co_0.85_Se electrodes in 2 M KOH electrolyte. The working electrodes of the two electrode system were fabricated similarly to the three electrodes. The as-prepared slurry was spread on the rounded nickel foam mesh current collector with an area of 1 cm^2^ uniformly, and the coating was left in an oven at 65°C overnight. Afterwards, this was weighed and pressed into sheets under 13 MPa to ensure adherence between the active materials and the current collector. The total mass of each electrode was limited to 3.0–5.0 mg. Two different electrodes of the same or very close weight were selected for measurement.

Finally, the ASCs were assembled using lamella-like Bi_18_SeO_29_/BiSe as the negative electrode and flower-like Co_0.85_Se as the positive electrode with a separator (filter paper) and electrolyte solution; they were assembled into a sandwich cell construction (electrode/separator/electrode). To uniformly diffuse the KOH electrolyte solution into the pseudocapacitive material electrode, the separated and pseudocapacitive material electrodes were immersed in 2 M KOH electrolyte for 6 h and then assembled into the supercapacitor configuration.

### 2.6. Electrochemical testing

A CHI660D electrochemical workstation (Shanghai Chenghua Instrument Co. Ltd, China) was used in the three-electrode and two-electrode systems by cyclic voltammetry (CV), galvanostatic charge/discharge (GCD) and electrical impedance spectroscopy (EIS). The EIS measurement was measured at a frequency ranging from 10 mHz to 100 kHz with an impedance amplitude of ±5 mV in open circuit potentials. In addition, a LAND CT2001A cell tester (Wuhan Landian electronics Co. Ltd, China) with a computer controlled system was used for the cycle-life stability test.

In the three-electrode system, the gravimetric capacitances of the Bi_18_SeO_29_/BiSe or Co_0.85_Se sample were calculated from the charge–discharge curves based on the following equation:

2.1 $$C_{s}=\frac{I\Delta t}{m\Delta V},$$

where *C*_s_ corresponds to the specific capacitance (F g^–1^), *I* represents the discharge current (A), Δ*t* represents the discharge time (s), *m* refers to the total mass (g) and Δ*V* refers to the voltage change (V) of the Bi_18_SeO_29_/BiSe or Co_0.85_Se.

For the two-electrode cells of ASCs, it was calculated by the equation

2.2 $$Q=C_{m}\times \Delta V\times m.$$

To obtain *Q*^+^ = *Q*^−^, as well as to make use of the largest voltage window, the mass ratio of the positive and negative electrodes are acquired on the basis of the following equations [33]:

2.3 $$\begin{array}{r} \frac{m^{+}}{m^{-}}=\frac{C_{m}^{+}\times \Delta V^{+}}{C_{m}^{-}\times \Delta V^{-}}, \end{array}$$

2.4 $$\begin{array}{r} C_{\mathrm{cell}}=\frac{I\Delta t}{m\Delta V}, \end{array}$$

2.5 $$\begin{array}{r} E=C_{\mathrm{cell}}\Delta V^{2}\times \frac{1000}{2\times 3600} \end{array}$$

2.6 $$\begin{array}{r} \;\text{and}\;P=E\times \frac{3600}{\Delta t}, \end{array}$$

where $C_{m}^{+}$ and $C_{m}^{-}$ are the specific capacitances (F g^–1^) , and Δ*V^+^* and Δ*V*^−^ represent the voltage ranges (V) of the Co_0.85_Se and Bi_18_SeO_29_/BiSe electrodes, respectively. *C*_cell_ denotes the specific capacitance (F g^–1^) of the ASC device, *I* the discharge current (A), Δ*t* the discharge time, *m* the total weight (g) of the two electrodes, Δ*V* the voltage window (V), *E* (Wh kg^–1^) the energy density and *P* (W kg^–1^) the power density [34].

## 3. Results and discussion

### 3.1. Negative electrode

**Figure 1.**
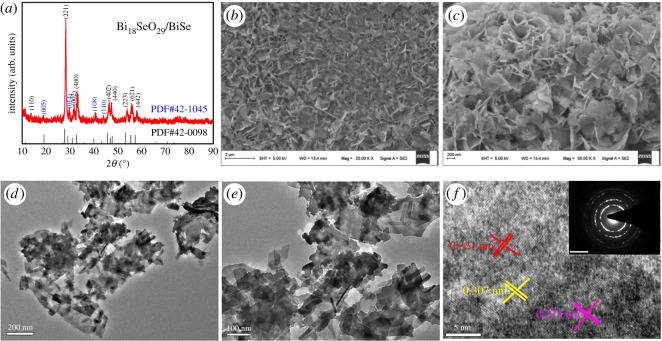
(*a*) XRD pattern of Bi_18_SeO_29_/BiSe nanosheets; (*b*,*c*) SEM and (*d*,*e*) TEM images of Bi_18_SeO_29_/BiSe nanosheets at various magnifications; (*f*) high-resolution TEM image of the Bi_18_SeO_29_/BiSe nanosheets.

**Figure 2.**
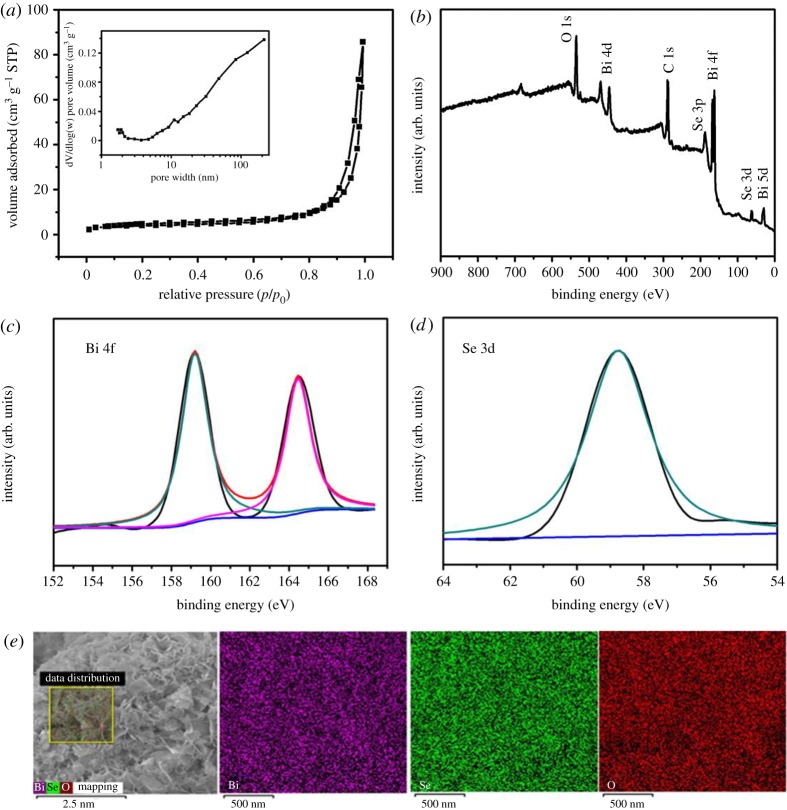
(*a*) Nitrogen adsorption–desorption isotherms and (inset) pore size distribution of Bi_18_SeO_29_/BiSe. (*b*) XPS of Bi_18_SeO_29_/BiSe; (*c*) high-resolution XPS spectra of Bi 4f peak; (*d*) high-resolution XPS spectra of Se 3d peak and (*e*) the corresponding element mapping images (selected from the square region) for the bismuth, selenium and oxygen of Bi_18_SeO_29_/BiSe nanosheets.

The purity and crystallinity of negative electrode Bi_18_SeO_29_/BiSe were investigated using powder XRD, as displayed in figure 1*a*; the peaks can be indexed to the hexagonal Bi_18_SeO_29_ phase (JCPDS card no. 42-0098) and the BiSe phase (JCPDS card no. 42-1045) from the XRD pattern. The strong diffraction peaks at 2*θ* angles of 27.77, 31.00, 32.74, 45.70, 46.97, 53.16, 55.45 and 57.34 could be readily indexed to the (221), (002), (400), (402), (440), (223), (621) and (442) planes of Bi_18_SeO_29_; the other diffraction peaks at 2*θ* angles of 19.16, 29.09, 39.98 and 43.28 can be indexed to the (005), (014), (108) and (110) planes of BiSe, respectively. The microstructure and morphology of Bi_18_SeO_29_/BiSe were investigated by SEM and TEM analyses. As indicated in figure 1*b*, the Bi_18_SeO_29_/BiSe exhibits a large amount of interconnected and ultrathin nanosheet structure. The high-resolution SEM image (figure 1*c*) gives a clear view of the nanosheets, consisting of abundant thin one-dimensional lamella-like structures which are well aligned together with intertwined nanosheet subunits; this can enable the easy flow and transfer of electrons and ions in the nanosheet structure. The unique structure of Bi_18_SeO_29_/BiSe can be further proved by TEM (figure 1*d*,*e*), in which are clearly displayed the thin layers with randomly intertwined nanosheet structure. Moreover, the high-resolution TEM image and the corresponding selected area electron diffraction (SAED), as described in figure 1*f*, demonstrate the polycrystalline nature of the nanosheets and illustrate clear lattice fringes, which can be assigned to the following crystal planes: 0.321 nm for Bi_18_SeO_29_ (221), 0.307 nm for BiSe (014) and 0.273 nm for Bi_18_SeO_29_ (400). A SAED image is displayed in figure 1*f* inset; the diffraction rings from inside to outside were indexed to the (221), (014), (108), (402) and (440) planes of the hexagonal Bi_18_SeO_29_/BiSe, which exhibits a crystalline characteristic. The clear lattice fringes and SAED are in agreement with the aforementioned XRD results. Although with some reservation, an approximate electrochemically active surface area was calculated from the Brunauer–Emmett–Teller method (BET) [35]. The BET surface area of the Bi_18_SeO_29_/BiSe is 19.35 m^2^ g^–1^, as shown in figure 2*a*, which is similar to that of pure Co_3_O_4_ material (18.5 m^2^ g^–1^) [36] and to that of emeraldine-di-hydrogen sulfate (22.1 m^2^ g^–1^) [37]. The electrochemically active surface area can facilitate intercalation and de-intercalation of electrolyte ions.

To further confirm the chemical element compositions and the surface valence state information, Bi_18_SeO_29_/BiSe was evaluated by the XPS technique and EDS, as presented in figure 2. In the XPS survey spectrum of Bi_18_SeO_29_/BiSe, the elements of Bi, Se and O as well as C can be clearly identified (figure 2*b*). Figure 2*c* shows the high-resolution spectrum of the Bi 4f region, which exhibits two asymmetrical main signals corresponding to the deconvolution of Bi 4f7/2 (159.28 ± 0.3 eV) and Bi 4f5/2 (164.45 ± 0.3 eV) with a spin–orbit splitting of about 5.4 eV [38]. The binding energies of 159.28 eV and 164.45 eV can be attributed to the Bi^3+^ ion [39]. From the significant dispersion of the binding energy, it can be demonstrated that bismuth forms a mixture rather than pure metal oxide [40]. Further, the core-level spectrum of the Se 3d region is shown in figure 2*d*; the binding energy of 58.7 eV can be assigned to oxidized Se (SeO_X_), which is in good agreement with previously reported values [30]. In addition, to verify the elemental distribution, Bi_18_SeO_29_/BiSe was further characterized by EDS (figure 2*e*); the corresponding element mapping images can unambiguously confirm the homogeneous distribution of bismuth, selenium and oxygen in the nanomaterial.

**Figure 3.**
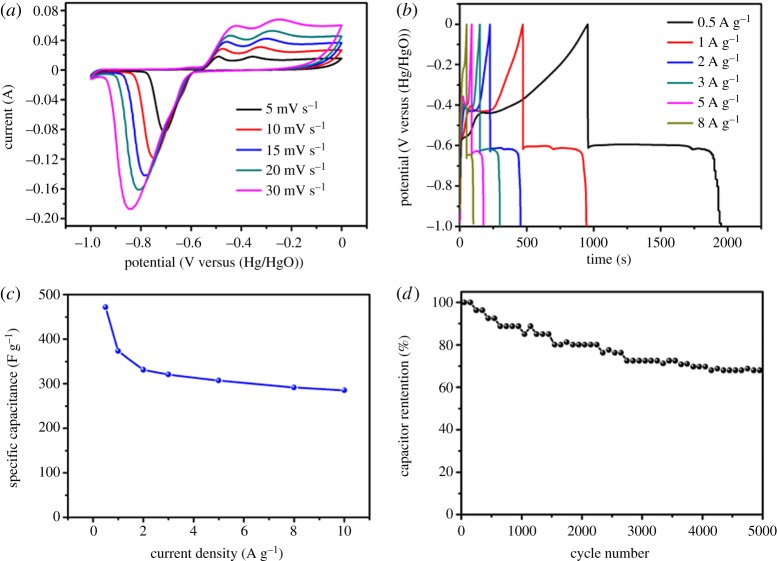
The electrochemical properties of synthesized Bi_18_SeO_29_/BiSe electrode materials via a three-electrode system: (*a*) CV curves at different scan rates; (*b*) GCD curves with various current densities; (*c*) with the increase in current density the changes in specific capacitance changes; and (*d*) cycling stability of the Bi_18_SeO_29_/BiSe electrode material at a current density of 2 A g^–1^.

The electrochemical behaviour of Bi_18_SeO_29_/BiSe nanosheets was first studied by CV and GCD techniques with a three-electrode system in 2 mol l^–1^ KOH aqueous electrolyte. Figure 3*a* displays the CV curves of the Bi_18_SeO_29_/BiSe nanosheets as the negative electrode at different scan rates ranging from 5 to 30 mV s^–1^ in the potential window of −1 to 0 V. A pair of redox peaks can be clearly observed in the CV curves of the Bi_18_SeO_29_/BiSe composite at different scan rates, showing it has typical Faradaic pseudocapacitance behaviour. In detail, the oxidation peaks were seen in forward CV scans, while the reduction peaks were seen in the reverse CV scans. In highly alkaline solution, hydroxide ions are generated naturally, which are likely to gravitate towards the cathode at high potentials Thus, the reduction peak at 0.7 V corresponds to Bi^3+^ transformed into $\text{Bi( OH}{{\text{)}}_{2}}^{+}$ in the reduction process. The oxidation peaks at about −0.48 V and −0.35 V correspond to Bi^3+^/Bi^0^ transformation. Those peaks are also seen in some other studies [27,41,42]. It may be catalysed by the induction or oxidation of some unconverted Bi^0^ during the reduction process, and the process has the following reaction [27,43]:

$$\begin{array}{rl} & \text{B}{\text{i}}^{3+}+2\text{O}{\text{H}}^{-}\to \text{Bi(OH}{{\text{)}}_{2}}^{+}\\ & 2\text{Bi(OH}{{\text{)}}_{2}}^{+}+\;2\text{O}{\text{H}}^{-}\to \text{B}{\text{i}}_{2}{\text{O}}_{3}+3{\text{H}}_{2}\text{O}\\ & \text{B}{\text{i}}_{2}{\text{O}}_{3}+2\text{O}{\text{H}}^{-}\to 2\text{Bi}{{\text{O}}_{2}}^{-}+{\text{H}}_{2}\text{O}\\ & \text{B}{\text{i}}_{18}\text{Se}{\text{O}}_{29}/\text{BiSe }+2\text{O}{\text{H}}^{-}\to \text{B}{\text{i}}_{18}\text{Se}{\text{O}}_{30}\text{H/BiSeOH}+{\text{e}}^{-}\\ & \text{Bi}{{\text{O}}_{2}}^{-}+{\text{e}}^{-}\to \text{Bi}{{\text{O}}_{2}}^{2-}\\ & \text{Bi}{{\text{O}}_{2}}^{2-}+2{\text{H}}_{2}\text{O}\to \text{Bi}{{\text{O}}_{2}}^{-}+4\text{O}{\text{H}}^{-}+\text{B}{\text{i}}^{0}\\ & \text{B}{\text{i}}^{0}\to \text{B}{\text{i}}_{\text{met}} \end{array}$$

Here, Bi^0^ are active atoms and Bi_met_ is the metal bismuth.

There are characteristic redox peaks of the electrode observed from −1 to 0 V which correspond to the reversible intercalation/deintercalation of OH^–^ ions occurring in the Bi_18_SeO_29_/BiSe bulk and at the interface of Bi_18_SeO_29_/BiSe and electrolyte to increase the discharge time.

From the CV curve, we can see that the shape remains similar even at high scan rates, which indicates that the Bi_18_SeO_29_/BiSe electrode material has excellent capacitance behaviour. And at higher sweep rates, the higher/lower potentials the reduction and oxidation peak potentials move, because the ions may only be immersed in the surface of the material; however at a lower sweep rates, ions can be effectively diffused into the inner active sites. As the lower scan rates are provided for a longer period of time, the active site interacts with the ions better. So it is obvious that as the scan rate increases, the specific capacitance will be reduced. The various current density GCD curves of the Bi_18_SeO_29_/BiSe electrode are presented in figure 3*b*; the charge/discharge curve reveals the pseudocapacitance behaviour. In the discharge process, there is a kink in the curve, which is perhaps some of the untransformed Bi^0^ oxidation platforms during the reduction process. These results are consistent with that consequence of the CV curves. As exhibited in figure 3*c*, the specific capacitances of the Bi_18_SeO_29_/BiSe electrode at various current densities of 0.5, 1, 2, 3, 5, 8 and 10 A g^–1^ are 471.3, 373, 331.2, 320.4, 307, 291.2 and 285 F g^–1^, respectively. This indicates outstanding rate capability [44]. This phenomenon is also present in transition metal sulfides and oxides/hydroxides, which can be attributed to the diffusion effect [45,46]. To evaluate the cycling stability, the GCD cycling of Bi_18_SeO_29_/BiSe was observed at a current density of 2 A g^–1^ (figure 3*d*), which indicates that Bi_18_SeO_29_/BiSe has good cycling stability with about 68% capacitance retention after 5000 cycles in 2 M KOH electrolyte. This result makes Bi_18_SeO_29_/BiSe a promising candidate as an advanced electrode material for supercapacitor application.

### 3.2. Positive electrode

**Figure 4.**
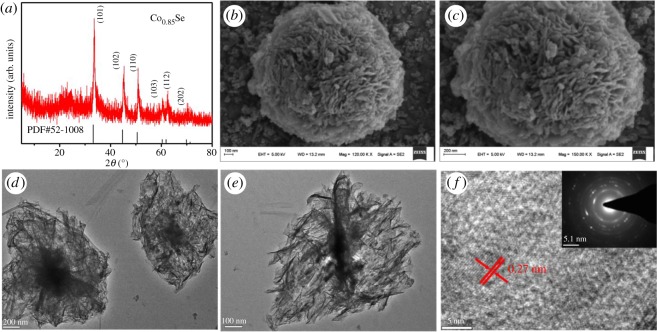
(*a*) XRD pattern of Co_0.85_Se nanosheets; (*b*,*c*) SEM and (*d*,*e*) TEM images of Co_0.85_Se nanosheets at various magnifications; (*f*) high-resolution TEM image of the Co_0.85_Se nanosheets.

The Co_0.85_Se was characterized in detail. From the XRD pattern displayed in figure 4*a*, the diffraction peaks can be indexed to Co_0.85_Se with the hexagonal structure (JCPDS card no. 52-1008) readily. The pure phase and sharp peaks show that Co_0.85_Se has high crystallinity. The strong diffraction peaks at 2*θ* = 33.3, 44.7 and 50.6 unequivocally correspond to the (101), (102), and (110) planes of Co0.85Se, respectively. The morphology characteristic features of the Co_0.85_Se sample were analysed via SEM. The SEM images (figure 4*b*) display a particular flower-like morphology, which is combined with the subunits of the nanosheet. The high-resolution SEM image (figure 4*c*) clearly shows a flower-like 3D microstructure, which could facilitate penetration of the electrolyte into the material and also increase the electrochemical reactions of the active sites. Further, the microstructure was also described by the corresponding TEM and high-resolution TEM images. As can be seen in figure 4*d*–*f*, Co_0.85_Se is constituted of many thin nanosheets, which are consistent with those in SEM. From figure 4*f*, well-defined lattice fringes are clearly seen with an interplanar spacing of 0.27 nm. This corresponds to the separation between (101) lattice planes of Co_0.85_Se. In the SAED pattern shown in the inset of figure 4*f*, the diffraction rings could be easily indexed to Co_0.85_Se with the hexagonal (101), (102), (110) and (103) planes from inside to outside, suggesting that the nanosheet is of polycrystalline nature [31], which shows good agreement with the XRD pattern and can be indexed to the structure.

**Figure 5.**
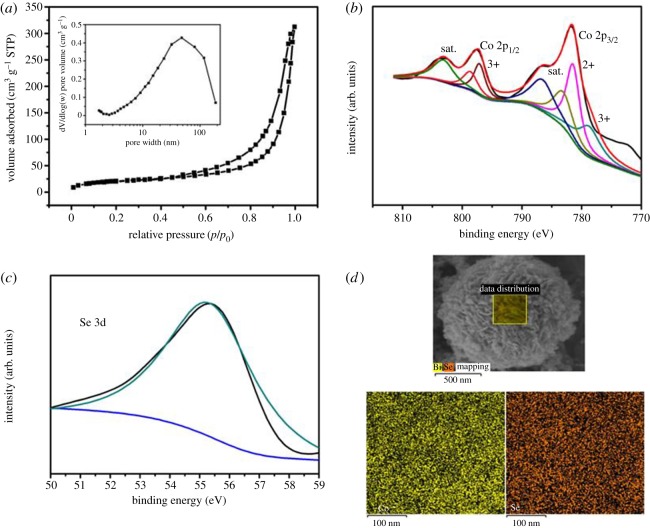
(*a*) Nitrogen adsorption–desorption isotherms and (inset) pore size distribution of Co_0.85_Se; (*b*) high-resolution XPS spectra of Co_2p1/2_ and Co_2p3/2_ peak; (*c*) high-resolution XPS spectra of Se 3d peak; and (*d*) the corresponding element mapping images (selected from the square region) for the Co_0.85_Se nanosheets.

The specific surface area and pore size distribution of Co_0.85_Se were examined by N_2_ adsorption–desorption measurements. As displayed in figure 5*a* and inset, at a relative pressure of 0.9–0.99, the apparent N_2_ adsorption and the hysteresis loop indicate the coexistence of a minor fraction of mesopores/macropores, which is mainly due to the 3D voids between interconnected particles [47]. The specific surface area of Co_0.85_Se calculated by the multiple points BET method is 73.3 m^2^ g^–1^, which is larger than that of the previously reported Co_0.85_Se electrode materials; Gong *et al*. described a specific surface area of 26.44 m^2^ g^–1^ [48] and Yang *et al*. described 59 m^2^ g^–1^ [31]. A mass of the 3D porous structure will favour the penetration of electrolyte and the rapid transmission of electrons, which will improve the electrochemical properties of the Co_0.85_Se electrode material.

The XPS and EDS techniques were used to evaluate the surface valence state information and the chemical element compositions of Co_0.85_Se (figure 5*b*–*d*), mainly exhibiting the Se 3d and Co 2p peaks. Figure 5*b* shows the Co 2p_3/2_, 2p_1/2_ and two satellite peaks (marked as ‘sat.’). The Co^3+^ 2p_3/2_, Co^2+^ 2p_3/2_ and Co^2+^ 2p_1/2_ are corresponding to binding energies of 779.0, 780.8 and 797.2 eV, respectively. The two spin–orbit doublets characteristic of Co^2+^ and Co^3+^ are considered and consistent with the previously reported values [37]. Furthermore, figure 5*c* clearly displays the core region of the binding energy of 55.4 eV corresponding to the Se 3d spectrum, and this approached the reported value [49]. The results show that the synthesized Co_0.85_Se electrode material consisted of Co^2+^, Co^3+^ and Se^2^, which is consistent with the formula Co_0.85_Se. Furthermore, the Co_0.85_Se electrode material is synthesized by element mapping images of cobalt and selenium in figure 5*d*, in which it is obvious that the elemental distributions are very uniform.

**Figure 6.**
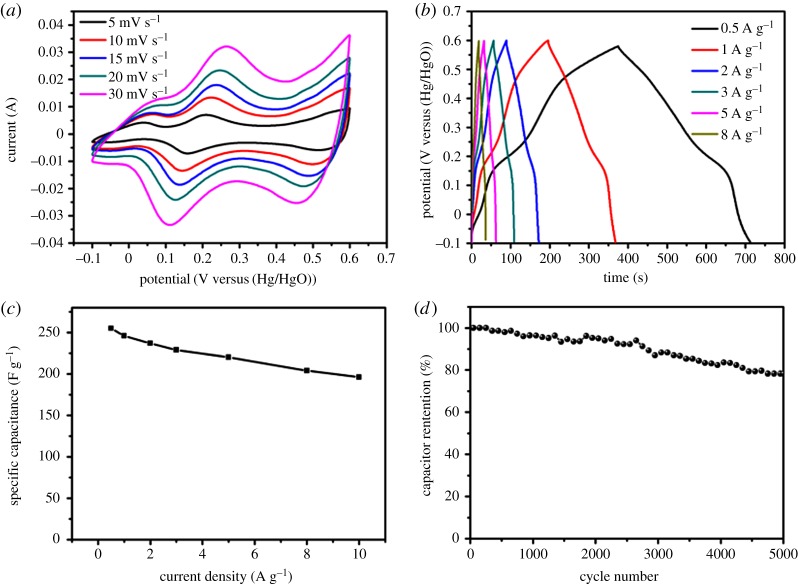
The electrochemical properties of synthesized electrode Co_0.85_Se materials in a three-electrode system: (*a*) CV curves with various scan rates; (*b*) GCD curves at different current densities; (*c*) the change of specific capacitance with the increase in current density; and (*d*) cycling stability of Co_0.85_Se electrode material at a current density of 2 A g^–1^.

Electrochemical behaviours of the Co_0.85_Se electrode material were investigated by CV and GCD measurements with 2 M KOH at a voltage window between −0.1 and 0.6 V (versus Hg/HgO) in a standard three-electrode system. From figure 6*a*, it is clear that the area surrounded by the CV curve of Co_0.85_Se exhibits two pairs redox peaks, which might be attributed to standard Faradaic pseudocapacitance behaviour. The two pairs ascribed to the reversible redox reaction can occur according to [50]

$$\begin{array}{rl} \text{C}{\text{o}}_{0.85}\text{Se }+\text{O}{\text{H}}^{-} & \to \text{C}{\text{o}}_{0.85}\text{SeOH}+{\text{e}}^{-}\\ \text{C}{\text{o}}_{0.85}\text{SeOH}+\text{O}{\text{H}}^{-} & \to \text{C}{\text{o}}_{0.85}\text{SeO}+{\text{H}}_{2}\text{O}+{\text{e}}^{-} \end{array}$$

Figure 6*a* describes the typical CV curves of the Co_0.85_Se nanosheets at various scan rates from 5 to 30 mV s^–1^, from which, even at a high scan rate of 30 mV s^–1^, two pairs of reversible redox peaks can be obviously observed. In addition, due to the presence of polarization, with the increase of the scan rate the position of the redox peak gradually changes [50]. The GCD test results are presented in figure 6*b* to analyse the charge storage capacity of the electrode material. Because the redox reaction occurs at the electrode interface by the desorption and adsorption of the hydroxyl ion in the alkaline electrolyte [51], it is obvious that each charge–discharge curve indicates pseudocapacitive performance at current densities from 0.5 to 8 A g^–1^, and deviates from the EDLC linear curve. All of the charge–discharge curves are almost symmetrical, which reveals that Co_0.85_Se has excellent capacitive behaviour. As displayed in figure 6*c*, the specific capacitance of Co_0.85_Se is calculated to be 255, 246, 237, 229, 220, 204 and 196 F g^–1^ at various current densities from 0.5 to 10 A g^–1^, exhibiting an outstanding rate capability. The stability of the Co_0.85_Se electrode material was tested using the charging and discharge technique at a current density of 2 A g^–1^. Finally, it is seen to retain about 78% of its initial capacitance after 5000 cycles, indicating that the Co_0.85_Se positive electrode material possesses outstanding cycling stability.

### 3.3. Asymmetric supercapacitor

**Figure 7.**
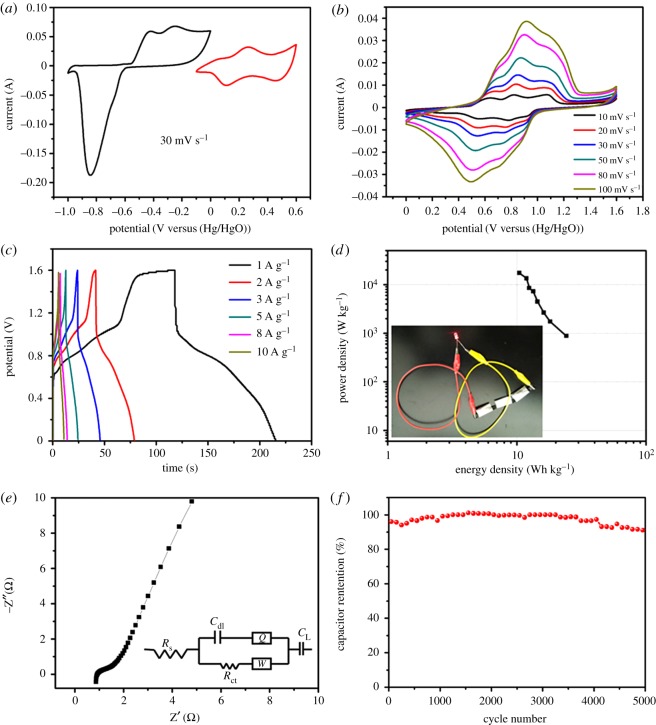
(*a*) CV curves of the Bi_18_SeO_29_/BiSe and Co_0.85_Se electrodes via a two-electrode cell at 30 mV s^–1^ in 2 M KOH; (*b*) CV curves of the ASC device with different scan rates; (*c*) GCD curves at different current densities; (*d*) Ragone plot related to energy and power densities of the ASC; (*e*) Nyquist plot of the ASC (inset shows the equivalent circuit model); (*f*) cycling stability of the Bi_18_SeO_29_/BiSe//Co_0.85_Se ASC in a two-electrode cell.

In practical applications, to further evaluate the electrochemical performance of the electrode materials, we assembled an ASC, in which Bi_18_SeO_29_/BiSe and Co_0.85_Se worked as the negative and positive electrode materials in 2 M KOH electrolyte. To ensure the electrochemical stability of the two-electrode cell with the acquired optimized potential window, the CVs of Bi_18_SeO_29_/BiSe (black) and the Co_0.85_Se (red) were tested in 2 M KOH electrolyte at 30 mV s^–1^. As displayed in figure 7*a*, the stable potential range of Co_0.85_Se is between 0 and 0.6 V and the capacitive behaviour of Bi_18_SeO_29_/BiSe is between −1 and 0 V. Therefore, the operating cell voltage can be optimized to 1.6 V in 2 M KOH aqueous solution for the ASC of Bi_18_SeO_29_/BiSe//Co_0.85_Se (figure 7*b*). In the design of the asymmetric cell, the balance of the charge stored between the positive and negative electrodes was necessary and it was calculated by equations (2.2) and (2.3). The CV curves of the Bi_18_SeO_29_/BiSe//Co_0.85_Se ASC device were analysed at various scan rates (between 10 and 100 mV s^–1^) with an operating voltage of 1.6 V. Figure 7*b* clearly shows that three pairs of redox peaks can be attributed to the redox reactions of Bi_18_SeO_29_/BiSe and Co_0.85_Se in 2 M KOH electrolyte. In addition, all curves show similar shapes; meanwhile changes in anode and cathode peaks even at high scan rate of 100 mV s^−1^ of the ASC are small, indicating outstanding reversibility of the ASC. Figure 7*c* exhibits the GCD curves of the Bi_18_SeO_29_/BiSe//Co_0.85_Se ASC device at different current densities (ranging from 0.5 to 8 A g^–1^), and this proves the coexistence of the oxidation of anions and surface adsorption of ions in this ASC device. An advanced Ragone diagram (energy density versus power density) of the Bi_18_SeO_29_/BiSe//Co_0.85_Se ASC device is obtained from the GCD data (figure 7*c*) as revealed in figure 7*d*, according to equations (2.5) and (2.6). The device exhibits a high energy density that reaches 24.2 Wh kg^–1^ at an outstanding power density of 871.2 W kg^–1^ at a current density of 1 A g^–1^. Even when the current density is as high as 15 A g^–1^, the energy density still remains at 11.9 Wh kg^–1^ with an excellent power density of 13 387.5 W kg^–1^. The energy density of the Bi_18_SeO_29_/BiSe//Co_0.85_Se ASC device is shown in figure 7*d*. To verify the feasibility of energy supply for the Bi_18_SeO_29_/BiSe//Co_0.85_Se ASC, the three-tandem cell group can light up a red light-emitting diode (figure 7*d*, inset). Therefore, the excellent high energy density indicates that it has great potential as a supercapacitor.

The facilitated ion or electron transport kinetics of the Bi_18_SeO_29_/BiSe//Co_0.85_Se ASC device was investigated by EIS. The Nyquist plot of the ASC device and the corresponding equivalent circuit are shown in figure 7*e* and in the inset of figure 7*e*, respectively. It consists of three different parts at a distinct frequency range: high frequencies, an unfinished semicircular part; the middle frequencies, an inclined portion in the curve (about 45°); and the low frequencies, the linear part. The high frequency of the real axis intercept indicates the internal resistance (*R*_s_), which is the sum of the large amount of electrolyte resistance, the contact resistance of the electrode/electrolyte interface and the intrinsic resistance of the electrode active material [52]. The charge transfer resistance (*R*_ct_) is the diameter of the semicircle which is related to the charge transfer at the electrode/electrolyte interface and thus produced a Faraday reaction [46]; in the middle frequencies, the 45° slope of the line is called Warburg impedance (*Z*_w_), which is caused by the diffusion process of the electrolyte [53]. Moreover, *C*_L_ and *C*_dl_ represent the limit capacitance and the double-layer capacitance [54]. As can be seen from the fitted results, a Bi_18_SeO_29_/BiSe//Co_0.85_Se ASC device not only has a low *R*_s_ (0.88 Ω cm^2^), but also possesses a small *R*_ct_ (1.87 Ω cm^2^), as well as a low Warburg resistance (0.017 Ω cm^2^). The values demonstrate that the electrolyte permeation and ion diffusion into the pore structure are very easy, and these values may belong to electrode materials with special structure. On the one hand, Bi_18_SeO_29_/BiSe is an ultrathin nanosheet, and it can provide an efficient pathway for charge transportation; on the other hand, Co_0.85_Se material has a 3D high surface area, for adsorbing ions to provide abundant electrical active sites. These results can facilitate transport of the electrolyte. The cyclic stability of the Bi_18_SeO_29_/BiSe//Co_0.85_Se ASC device is evaluated by the GCD process with a current density of 2 A g^–1^ after 5000 cycles and an operating voltage of 0–1.6 V (figure 7*f*). Owing to the full activation of the Bi_18_SeO_29_/BiSe and Co_0.85_Se materials, the specific capacitance increases at the beginning, and even after 1500 cycles, the degradation of the active material was not observed with maintenance of 100% initial capacity. Thus, excellent cycling performance is shown. Finally, the ASC device shows only a slight decrease in specific capacitance (about 93% of the initial specific capacitance retention after 5000 cycles), indicating excellent cycle stability. The above results mean that the high-performance ASC with excellent stability may be a candidate for energy storage devices in future electronic application.

## 4. Conclusion

In summary, the negative Bi_18_SeO_29_/BiSe and positive Co_0.85_Se electrode materials are prepared through a simple one-step hydrothermal method without any template and surfactant. Bi_18_SeO_29_/BiSe and Co_0.85_Se have high specific capacitance (471.3 F g^–1^ and 255 F g^–1^ at 0.5 A g^–1^), high conductivity, outstanding cycling stability, as well as good rate capability. The all-pseudocapacitive electrodes fabricated Bi_18_SeO_29_/BiSe//Co_0.85_Se ASC device has excellent electrochemical performance with a good cycling stability (93% capacitance retention after 5000 cycles at a current density of 2 A g^–1^) and high energy density (about 24.2 Wh kg^–1^) as well as high power density (about 871.2 W kg^–1^) in aqueous electrolyte at a wide voltage window of 0–1.6 V. The results demonstrate that the electrode material preparation approach is easy and also the resulting ASC is promising as an energy-storage device.

## Data accessibility

This article has no additional data.
